# Design paper of the “Blood pressure targets in post-resuscitation care and bedside monitoring of cerebral energy state: a randomized clinical trial”

**DOI:** 10.1186/s13063-019-3397-1

**Published:** 2019-06-10

**Authors:** Simon Mölström, Troels Halfeld Nielsen, Carl H. Nordström, Christian Hassager, Jacob Eifer Møller, Jesper Kjærgaard, Sören Möller, Henrik Schmidt, Palle Toft

**Affiliations:** 1 Department of Anesthesiology and Intensive Care, Odense University Hospital, J. B. Winsløws Vej 4, 5000 Odense C, Denmark; 2 Department of Neurosurgery, Odense University Hospital, J. B. Winsløws Vej 4, Odense, 5000 Denmark; 3 Department of Cardiology, Odense University Hospital, J. B. Winsløws Vej 4, Odense, 5000 Denmark; 4 OPEN – Odense Patient data Explorative Network, University of Southern Denmark, Odense University Hospital and Department of Clinical Research, J. B. Winsløws Vej 9, Odense, 5000 Denmark; 5 The Heart Centre, Copenhagen University Hospital, Blegdamsvej 9, Copenhagen, 2100 Denmark

**Keywords:** Out-of-hospital cardiac arrest, Neuroprotection, Blood pressure, Microdialysis, Cerebral metabolism

## Abstract

**Background:**

Neurological injuries remain the leading cause of death in comatose patients resuscitated from out-of-hospital cardiac arrest (OHCA). Adequate blood pressure is of paramount importance to optimize cerebral perfusion and to minimize secondary brain injury. Markers measuring global cerebral ischemia caused by cardiac arrest and consecutive resuscitation and reflecting the metabolic variations after successful resuscitation are needed to assist a more individualized post-resuscitation care. Currently, no technique is available for bedside evaluation of global cerebral energy state, and until now blood pressure targets have been based on limited clinical evidence. Recent experimental and clinical studies indicate that it might be possible to evaluate cerebral oxidative metabolism from measuring the lactate-to-pyruvate (LP) ratio of the draining venous blood. In this study, jugular bulb microdialysis and immediate bedside biochemical analysis are introduced as new diagnostic tools to evaluate the effect of higher mean arterial blood pressure on global cerebral metabolism and the degree of cellular damage after OHCA.

**Methods/design:**

This is a single-center, randomized, double-blinded, superiority trial. Sixty unconscious patients with sustained return of spontaneous circulation after OHCA will be randomly assigned in a one-to-one fashion to low (63 mm Hg) or high (77 mm Hg) mean arterial blood pressure target. The primary end-point will be a difference in mean LP ratio within 48 h between blood pressure groups. Secondary end-points are (1) association between LP ratio and all-cause intensive care unit (ICU) mortality and (2) association between LP ratio and survival to hospital discharge with poor neurological function.

**Discussion:**

Markers measuring cerebral ischemia caused by cardiac arrest and consecutive resuscitation and reflecting the metabolic changes after successful resuscitation are urgently needed to enable a more personalized post-resuscitation care and prognostication. Jugular bulb microdialysis may provide a reliable global estimate of cerebral metabolic state and can be implemented as an entirely new and less invasive diagnostic tool for ICU patients after OHCA and has implications for early prognosis and treatment.

**Trial registration:**

ClinicalTrials.gov (ClinicalTrials.gov Identifier: NCT03095742). Registered March 30, 2017.

**Electronic supplementary material:**

The online version of this article (10.1186/s13063-019-3397-1) contains supplementary material, which is available to authorized users.

## Background

In comatose patients resuscitated from out-of-hospital cardiac arrest (OHCA), anoxic brain injuries remain the leading cause of death [1]. The overall mortality, though improved considerably over the last decade, continues to be significant and in most countries is as high as 90% [2, 3]. Despite successful resuscitation and admission to an intensive care unit (ICU), the in-hospital mortality is reported to be 30–50% [4, 5]. Functional favorable outcomes among OHCA survivors remain low, and global anoxic brain injury represents a major mechanism of long-term disability [6, 7]. During the post-resuscitative phase, the most significant clinical challenge is to limit secondary brain injury. Targeted temperature management (TTM) targeting 33–36 °C may mitigate cerebral ischemia–reperfusion injury and is recommended in current international guidelines [8–10]. However, managing post-cardiac arrest patients is far more complex than TTM alone, and brain-directed therapies include maintenance of normal oxygenation, ventilation, hemodynamic support to optimize cerebral perfusion, and glycemic control [10, 11].

Reduced cerebral blood flow (CBF), which is mainly dependent upon mean arterial blood pressure (MAP), can result in brain ischemia and enhance secondary brain injury after cardiac arrest. Arterial hypotension may exacerbate brain injury following cardiac arrest because of ongoing cerebral hypoperfusion. Cerebral autoregulation is frequently impaired after cardiac arrest [12, 13], and brain perfusion declines when the MAP falls below 80 to 100 mm Hg. Thus, the generally recommended MAP of above 65 mm Hg [11] to reverse the acute shock state may be inadequate to maintain adequate cerebral perfusion.

When considering blood pressure goals, clinicians should balance the metabolic needs of an ischemic brain with the potential for overstressing a decompensated heart. Post-cardiac arrest myocardial dysfunction, caused by coronary infarction and ischemia–reperfusion injury, is common after CA and resuscitation. Excessive vasopressor use may cause increased afterload and augmented oxygen consumption of the heart, thereby aggravating the myocardial damage [14]. High-dose vasopressor use has been associated with increased mortality [15].

Randomized control trials addressing specific MAP targets in post-resuscitation care have not yet been performed. This study addresses strategies for neuroprotection and will randomly assign a total of 60 unconscious resuscitated patients after OHCA in a double-blinded one-to-one fashion to low (mean 63 mm Hg) or high (mean 77 mm Hg) MAP target.

Secondary brain injury processes throughout the post-resuscitation period remain complex and involve numerous pathophysiological pathways that result from secondary ischemia and reperfusion injury. These include cerebral edema, inflammation, unbalanced CBF, and mitochondrial dysfunction [16]. Markers measuring global cerebral ischemia and reflecting the metabolic variations after resuscitation are needed for a more individualized post-resuscitation care and target-driven therapy to improve patient outcome [17, 18].

**Fig. 1:**
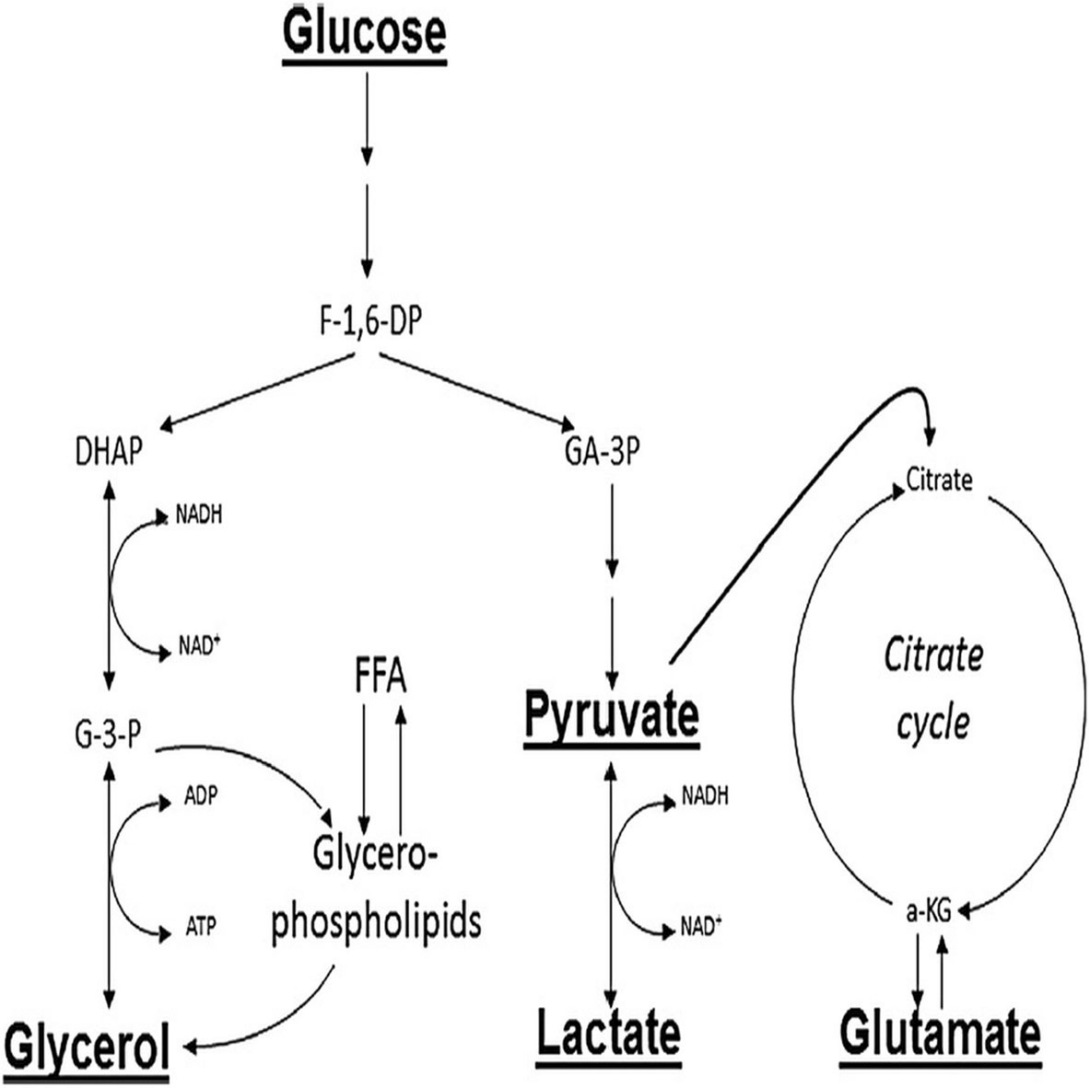
Schematic diagram of cerebral metabolism. Schematic illustration of cerebral intermediary metabolism is shown with a focus on the glycolytic pathway and its relation to glycerol, glycerophospholipids, and the citric acid cycle. *Abbreviations*: *α-KG* α-ketoglutarate, *DHAP* dihydroxyacetone-phosphate, *F-1,6-DP* fructose-1,6-diphosphate, *FFA* free fatty acid, *G-3-P* glycerol-3-phosphate, *GA-3P* glyceraldehyde-3-phosphate. Underscored metabolites are obtained at the bedside with enzymatic techniques.

Microdialysis (MD) allows biochemical variables of the extracellular interstitial fluid to be monitored continuously and provides data on substrate supply and metabolism at the cellular level in the brain [19]. Cerebral energy metabolism is strictly aerobic, and the parameters monitored (glucose, pyruvate, lactate, glutamate, and glycerol) are closely linked (Fig. 1) [20]. Under clinical conditions, the cerebral cytoplasmic redox state is conventionally evaluated from the lactate-to-pyruvate (LP) ratio obtained from intracerebral MD and bedside biochemical analysis. Compromised energy metabolism will cause a shift in the cytoplasmic redox state that is immediately reflected in an increase of the LP ratio [21].

Clinical MD monitors the supply of glucose and its metabolism via glycolysis to pyruvate, which under oxidative conditions enter the tricarboxylic acid cycle. During hypoxic conditions or if mitochondrial function is compromised, pyruvate is metabolized to lactate. Therefore, the LP ratio is used as a marker of aerobic versus “anaerobic” metabolism not requiring oxygen [22]. High LP ratio is considered a robust indicator of anaerobic metabolism and the redox status of the tissue and is an independent predictor of mortality and unfavorable outcome in traumatic brain injury among patients monitored with MD [23–27]. An increased LP ratio could result from a failure of oxygen delivery (ischemic hypoxia) or no ischemic causes (e.g., mitochondrial dysfunction) [28]. An increase in the LP ratio in the presence of low pyruvate (and low oxygen pressure) indicates ischemia. In contrast, an increase in LP ratio in the presence of normal or high pyruvate (and normal oxygen pressure) indicates mitochondrial dysfunction. With intracerebral MD, the normal upper limit for the LP ratio of the human brain has been defined as 30 [22, 24]. Increased levels of glutamate and glycerol indicate hypoxia/ischemia and have been defined as indicators of excitotoxicity and cell membrane breakdown, respectively [19].

The LP ratio obtained from MD of cerebral venous blood may be a sensitive indicator of impending cerebral damage and secondary neurological deterioration and might play a critical role in detecting the early responses of post-resuscitation care. The use of global cerebral MD may potentially aid clinicians in providing individualized and tailored brain resuscitation strategies that prevent secondary brain injury and lead to improved survival and neurological outcomes after cardiac arrest.

Our group recently reported how it is technically simple and feasible to place an MD catheter in the jugular bulb and monitor biochemical variables related to global cerebral energy metabolism at the bedside during cardiac surgery [29]. In the study, we evaluated a new method of cerebral metabolic monitoring, namely MD of cerebral venous blood drainage. The cerebral venous blood drains mainly to the jugular bulbs. Most of the outflow is to either the left or right side, depending on dominance. The study concluded that metabolic monitoring in the central venous outflow (jugular bulb) is representative of the overall cerebral metabolism and can be used in the diagnosis of compromised global cerebral metabolism during cardiac surgery.

## Methods/Design

This study is a double-blinded, randomized, superiority clinical trial assessing the effect of different blood pressure levels on global cerebral metabolism in addition to TTM in adult comatose patients resuscitated from OHCA. Patients are enrolled at one Danish university hospital with a population background of about 1.3 million citizens for highly specialized cardiac care. The SPIRIT (Standard Protocol Items: Recommendations for Interventional Trials) 2013 Checklist, together with the Statement, facilitated with drafting of the trial protocol (Additional file 1).

### Aim

The study aims are to (a) investigate whether the LP ratio obtained by MD of the cerebral venous outflow reflects a derangement of global cerebral energy state after OHCA, (b) investigate the effect of higher MAP on global cerebral metabolism and LP ratio in comatose patients resuscitated from OHCA, and (c) investigate the correlation between LP ratio and neurological outcome (cerebral performance category, or CPC). The null hypothesis is that a higher MAP will not reduce LP ratio within 48 h compared with lower MAP.

### Trial procedure

#### Phase 1 (hospital admission to intervention onset)

Comatose patients after OHCA admitted to the hospital with sustained return of spontaneous circulation (ROSC) are eligible for screening. Acute computed tomography (CT) scan or acute coronary angiogram performed before ICU admission is also included in this phase. The inclusion window is 220 min from ROSC to screening. Patients eligible for the trial must comply with all inclusion criteria according to Table 1 at randomization. In accordance with Danish legislation, if no exclusion criteria are met, proxy informed consent is obtained from next of kin and from one independent medical doctor not involved with the trial.

**Table 1:** Inclusion and exclusion criteria

Inclusion criteria	
1. Age of at least 18 years	
2. Out-of-hospital cardiac arrest (OHCA) of presumed cardiac cause	
3. Sustained return of spontaneous circulation (ROSC), defined as ROSC when chest compressions have not been required for 20 consecutive minutes and signs of circulation persist	
4. Unconsciousness (Glasgow Coma Scale (GCS) score of less than 8) after sustained ROSC	
5. Target temperature management (TTM) is indicated.	
Exclusion criteria	
1. Conscious patient (GCS score of at least 8)	
2. Female of child-bearing potential, unless a negative human chorionic gonadotropin (hCG) test can rule out pregnancy within the inclusion window	
3. In-hospital cardiac arrest (IHCA)	
4. OHCA of presumed non-cardiac cause, such as after trauma, dissection/rupture of major artery or arrest caused by hypoxia (i.e., drowning, hanging, etc.)	
5. Known bleeding diathesis (medically induced coagulopathy does not exclude patient)	
6. Suspected or confirmed acute intracranial bleeding	
7. Suspected or confirmed acute ischemic stroke	
8. Unwitnessed asystole	
9. Known limitations in therapy and do-not-resuscitate order	
10. Known disease making 180-day survival unlikely	
11. Known pre-arrest cerebral performance category (CPC) score of 3 or 4	
12. More than 4 h (240 min) from ROSC to randomization	
13. Systolic blood pressure of less than 80 mm Hg in spite of fluid loading/vasopressor and/or inotropic medication and/or mechanical circulatory support*	
14. Temperature of less than 30 °C on admission	
15. Uncorrected blood glucose of less than 2.5 mmol/L at admission	

* If systolic blood pressure is recovering during the inclusion window the patient can be included

Enrollment of patients will be performed by the attending physician who is not involved in data collection or assessment. Randomization of blood pressure target was performed by a computerized algorithm using STATA (StataCorp LLC, College Station, TX, USA) in varying block sizes of 4, 8, and 12 patients. Baseline characteristics are obtained.

#### Phase 2 (intervention period)

OHCA patients are randomly allocated to either low- or high-blood pressure target during ICU stay: Allocation of patients resuscitated from OHCA to low- or high-blood pressure target during ICU stay):. The  first invasive blood pressure using the trial blood pressure module is marking the start of the intervention, most often coinciding with initiation of TTM. Interventions are considered emergency procedures, and study blood pressure measurement using the study blood pressure modules (HP/Philips M1006B Invasive Pressure module) is commenced as soon as possible after sustained ROSC, screening, and randomization. Study target blood pressure will be blinded. In half of the modules, the calibration factor will be adjusted in order to show the blood pressure measurements about 10% lower than the patients’ actual blood pressure at 100 mm Hg, and in half of the patients, the blood pressure measurements will be shown to be about 10% higher at 100 mm Hg. Targeting an MAP of 70 mm Hg during treatment in both groups will mean a blinded comparison of 63 and 77 mm Hg, both of which are within the usually acceptable ranges of blood pressure. A prospective, randomized, controlled clinical study has validated this method for double-blinded comparison of MAP targets in the ICU setting [30]. The intervention continues for as long as the patient has invasive blood pressure measurements.

##### Blood pressure management during ICU stay

MAP is increased by administration of (a) volume resuscitation until CVP is at least 10 mm Hg and distensibility index of the inferior vena cava is less than 12%, unless pulmonary edema is clinically apparent, and (b) norepinephrine (μg/kg per min solution) is administered in increments of 0.02 μg/kg per min until a dose of 0.2 μg/kg per min is reached. A higher dose will be used at the discretion of the attending physician; (c) dopamine (μg/kg per min solution) will be used in addition to norepinephrine at a maximal dose of 10 μg/kg per min.

As part of the routine hemodynamic monitoring, a pulmonary artery catheter (PAC) is inserted via the internal jugular or subclavian vein under ultrasound guidance. During ICU stay, cardiac index, mixed venous oxygen saturation (SvO_2_), central venous, and pulmonary pressures were measured continuously by PAC (CCOmbo PAC^®^, Edwards Lifesciences, Irvine, CA, USA) linked to the correct monitor (Vigilance II^®^, Edwards Lifesciences). These data, together with information on blood temperature, oxygen saturation, and MAP measured from a radial artery line, were transferred electronically to a computer with a 2-second time interval. If echocardiography, and thermodilution suggest that low cardiac output addition of an inotropic agent may be appropriate). The following agents may be considered: (a) milrinone (0.375–0.75 μg/kg per min) and (b) levosimendan (0.1 μg/kg per min). If an inotropic agent at maximal dose combined with vasopressors cannot maintain cardiac output, mechanical circulatory support may be considered.

##### Neuromonitoring during ICU stay

At ICU arrival, patients will be implanted with a jugular bulb MD catheter and monitored for 96 h or until arousal. MD samples are collected in microvials and analyzed every 1 h at the bedside. Regional cerebral oxygen saturation will be monitored by using bifrontal NIRS (Somanetics INVOS Cerebral Oximeter system). Right and left frontal regional oxygen saturation (rSO_2_) values will be recorded simultaneously during ICU stay, and values will be recorded every hour for 96 h or until arousal.

##### Mechanical ventilation and oxygenation during ICU stay

Patients will be mechanically ventilated, sedated, and when necessary paralyzed with neuromuscular blocking agents to reduce shivering and subsequent heat generation and energy consumption. The study is targeting normal oxygenation (13–14 kPa) during TTM and mechanical ventilation. Ventilation is adjusted, targeting normocapnia of partial pressure of carbon dioxide (paCO_2_) of 4.5–6.0 in all patients.

Serial arterial blood gas will be analyzed open-label by using commercially available equipment adjusted to 37 °C (alpha-stat). The TTM intervention period of 24 h commenced at the time of randomization by using an automated feedback device with temperature control to achieve a target core temperature of 36 °C. After TTM, patients are rewarmed to a core temperature of 37 °C with no more than 0.5 °C per hour. Phase 2 ends when sedation is withheld.

#### Phase 3 (from end of sedation to 72 h after OHCA)

Sedation is terminated after rewarming when the temperature is at least at 37 °C. Normothermia of 37 °C ± 0.5 °C is maintained until 72 h after the cardiac arrest as long as the patients are managed in the ICU and are unconscious. Nevertheless, weaning from ventilation will be attempted at the earliest possible time during this phase on the basis of typical procedures for discontinuation of mechanical ventilation. Blinded physicians perform a neurological evaluation of patients who remain in coma at 72 h or later after OHCA. The number of patients still comatose at 72 h after the end of TTM who underwent neurological prognostication will be reported. The number of patients who died before neurological prognostication and their presumed cause of death, including limitations in care and explanations for that, will be described. If judged by attending physicians to be indicated, electroencephalogram, somatosensory evoked potentials, and CT of the brain will be performed.

#### Phase 4 (end of intervention period to hospital discharge)

This starts when invasive blood pressure measurement is discontinued during the ICU stay.

Neurological outcome will be assessed at hospital discharge in accordance with the CPC scale [31, 32]: CPC 1, no neurological deficit; CPC 2, mild to moderate dysfunction; CPC 3, severe dysfunction; CPC 4, coma; and CPC 5, death. CPC scores of 1 and 2 are mostly considered “good” outcomes, and a CPC 3–4 “poor” outcomes. Physicians performing the CPC assessment will be blinded to patient’s LP ratio levels.

#### Phase 5 (hospital discharge/3 months after end of trial)

**Fig. 2:**
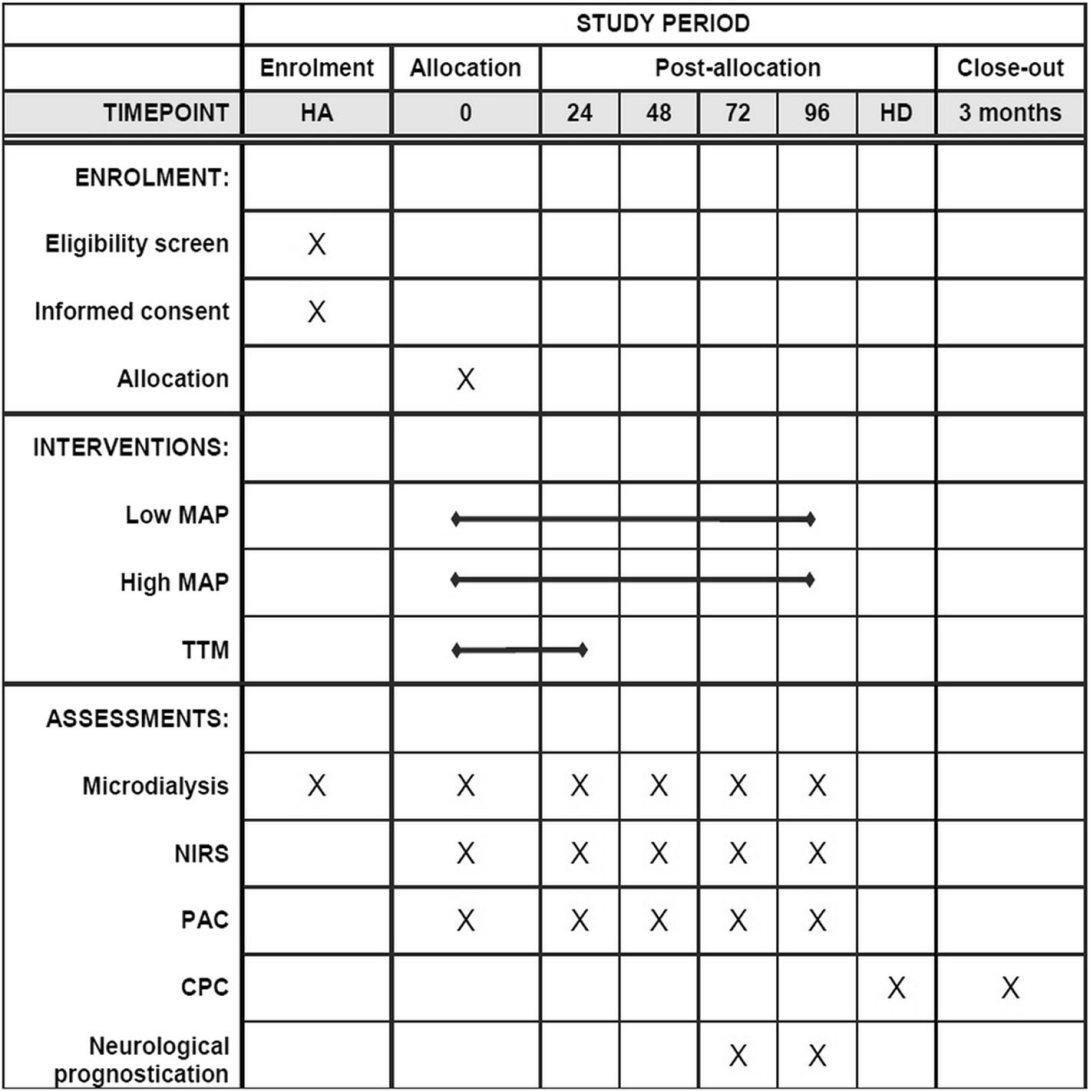
Overall schedule and time commitment for trial participants. *Abbreviations*: *CPC* cerebral performance category, *HA* hospital admission, *HD* hospital discharge, *MAP* mean arterial blood pressure, *NIRS* near infrared spectroscopy, *PAC* pulmonary artery catheter, *TTM* targeted temperature management.

Vital status and neurological status (CPC) were evaluated at 3 months after admission by clinical examination or telephone interview. Vital status will be evaluated at the end of the trial by using the Danish civil registration system. The SPIRIT timeline of progress through the phases of the parallel-randomized trial is shown in Fig. 2.

### Inclusion

Patients at least 18 years old with sustained ROSC after OHCA are eligible for inclusion if complying with all the criteria presented in Table 1.

### Bedside monitoring of cerebral energy microdialysis

Patients will be implanted with a jugular bulb MD catheter (CMA 67 IV 130 mm, MDialysis AB, Stockholm, Sweden) and monitored for 96 h or until arousal. The catheter allows energy-related metabolites to diffuse into the catheter reflecting accurate concentrations in the venous blood, CE marked according to the Medical Device Directive, 93/42/EEC. MD catheters are inserted in a retrograde direction into the jugular bulb, through a peripheral intravenous 17 GA cannula by using ultrasound guidance, as performed in a previous study [29]. The positioning of the catheter tip in the jugular bulb above the inlet of the common facial vein is confirmed on cranial CT scan in accordance with earlier practice [33]. MD pumps (CMA 106, MDialysis AB) at 0.3 μL/min perfuse MD catheters. The perfusates are collected in microvials, analyzed every 1 h for minimum 96 h by enzymatic photometric techniques, and presented at the bedside (Iscus, Mdialysis AB). The analyses include the biochemical variables regularly monitored during intracerebral MD: glucose, pyruvate, lactate, glutamate, and glycerol. Clinical data, radiology, electroencephalography, somatosensory evoked potential, and NIRS will be recorded.

### Logistics

The study is enrolling at one university hospital with experience in conducting clinical trials. All investigators have been qualified and are licensed in “good clinical practice” (GCP).

### Patient and public involvement

Patients were not involved in setting the research hypothesis or end-points, nor were they implicated in the design of the study. There are no plans to include patients in the dissemination of results, nor will we publish results directly to patients.

### End-points

The study focuses on the following end-points:

Primary end-point

1. Comparing mean LP ratio differences within 48 h between blood pressure groups and comparing individual LP ratio measurements using longitudinal analysis.

Secondary end-points

1. Association between LP ratio and all-cause mortality during ICU stay adjusted for blood pressure groups
2. Between-group difference in total duration (minutes) of cerebral desaturation defined as an absolute rSO_2_ of not more than 50% during ICU stay
3. Between-group difference within 48 h in lactate, pyruvate, glutamate, glycerol, and glucose measured in jugular bulb
4. Association between LP ratio and cardiac index adjusted for blood pressure groups
5. Association between LP ratio and survival to hospital discharge with poor neurological function (CPC 3–4) adjusted for blood pressure groups.

### Sample size estimation

The sample size was calculated on the basis of the primary hypothesis but taking into account only the mean LP ratio and not the individual measurements; this was due to insufficient information about interpretation variation in LP values; this implies a conservative error and hence the true power of the study is expected to be higher than in this calculation. The trial is designed as a randomized study, and we have chosen to power this study according to differences in the LP ratio. A difference of 30% between groups is defined as the minimal clinically relevant difference. Assuming an average LP ratio of less than 20 over 48 h in the high MAP group compared with more than 35 over 48 h in the low MAP group, we will need to include 46 patients in total to achieve a power of 0.9 using a patient-to-patient variation with standard deviation (SD) of 15 in LP ratio as motivated by an earlier study [29] in the power calculation and assuming normal distribution of LP ratio. To take possible higher patient-to-patient variation in this study as well as deviations from normality into account, we plan to include at least 60 patients in total. Defining an LP ratio of more than 30 averaged over 24 and 48 h for both groups, we will analyze the proportion of patients reaching that end-point in each group.

### Statistical analysis plan

The statistical analyses will be the following:

1. Analyses will be performed in accordance with the intention-to-treat principle and will take into account patients lost to follow-up.
2. A two-sided significance level of 0.05 will be applied to both primary and secondary end-points.
3. Unpaired *t* tests or Mann–Whitney *U* tests will be conducted for unpaired comparisons of numerical variables. A chi-squared or Fisher’s exact test will be conducted to examine differences between categorical variables.
4. Association between LP ratio and “poor” neurological outcome (CPC 3–4) will be assessed with chi-squared test and logistic regression. The overall expected rates of unfavorable neurological outcome (CPC 3–4) are 5–7%, observed in earlier studies [34, 35]. The multivariate logistic model will be adjusted for time to ROSC, arterial carbon dioxide partial pressure, baseline LP, and average LP ratio > 30 > 24 and 48 h as covariates.
5. Association between LP ratio and all-cause mortality will be assessed with chi-squared test and logistic regression.
6. Receiver operating characteristic (ROC) curves will be constructed to determine the sensitivity and specificity of the LP ratio levels at each time point (and the maximal LP ratio level recorded during the ICU period) for predicting outcomes.
7. Association between total duration (in minutes) of cerebral desaturation (rSO_2_ ≤ 50%) and unfavorable neurological outcome (CPC 3–4) and death will be assessed with chi-squared test and logistic regression.
8. Time from start of randomization to death in the two blood pressure groups will be assessed by using the Kaplan–Meier method, and group differences will be tested by log-rank test. Cox regression will be applied for adjusted comparisons and estimation of hazard ratios.
9. In regard to dynamic changes of MD variables in the two blood pressure groups, longitudinal models, applying linear mixed effects regression, will be used to account for repeated measurements of MD biomarkers across different patients over time.
10. In regard to dynamic changes of blood pressure, longitudinal models, applying linear mixed effects regression, will be used to account for repeated measurements of blood pressure across different patients over time.

All standard assumptions regarding statistical models will be checked. Statistical analysis will be performed by using STATA V.15 (StataCorp LLC).

### Trial profile

A flowchart of the study participants will be presented in accordance with the Consolidated Standards of Reporting Trials (CONSORT) diagram [36, 37].

### Baseline data

The predefined baseline variables are the following:

1. Sex
2. Age
3. Comorbidities (premorbid CPC, ischemic heart disease, heart failure, previous cardiac arrest, arterial hypertension, stroke, epilepsy, diabetes, chronic obstructive pulmonary disease, chronic hemodialysis, and alcoholism)
4. Previous percutaneous coronary intervention
5. Previous coronary artery bypass graft
6. Previous valvular surgery
7. Implantable cardioverter-defibrillator or pacemaker or both
8. Pre-hospital variables
  - Location of cardiac arrest
  - Bystander-witnessed arrest
  - Bystander cardiopulmonary resuscitation
  - Shockable primary rhythm
  - Time to basic life support
  - Time to advanced life support
  - Time to ROSC
9. Admission variables
  - First measured temperature
  - Glasgow Coma Scale score
  - Shock at admission
  - Presumed cause of cardiac arrest (cardiac/non-cardiac)
  - Acute myocardial infarction
  - Serum pH
  - Serum lactate
  - End-tidal carbon dioxide
  - Blood pressure
  - Result of angiogram
  - Cardiac troponin (TnI) and creatine kinase-muscle/brain (CK-MB).

Differences in baseline variables between blood pressure groups will be analyzed and presented in tables. Continuous variables will be presented as mean ± SD, and differences will be analyzed with the unpaired *t* test. In case of non-normally distributed data, continuous variables will be presented as median (interquartile range) and a *t* test will be applied following logarithmic transformation if the transformed data are normally distributed; alternatively, the non-parametric Mann–Whitney test will be applied. Categorical variables will be presented as counts and percentages and differences will be analyzed with the chi-squared test or Fisher’s exact tests if counts below 10 are observed.

## Discussion

The mortality of patients who are admitted in a comatose state to an ICU following successful resuscitation after cardiac arrest remains significant. The necessity for early and accurate prognostic predictors is important, especially since sedation and TTM might change the neurological examination and postpone the recovery of motor response for several days. Markers measuring global cerebral ischemia caused by cardiac arrest and consecutive resuscitation and reflecting the metabolic variations after successful resuscitation are urgently required to assist more personalized post-resuscitation care and prognostication.

Jugular bulb MD may provide a reliable global estimate of cerebral metabolic state and can be implemented as an entirely new and less invasive diagnostic tool for ICU patients after OHCA and has implications for early prognosis and treatment. The LP ratio assessed from MD of cerebral venous blood may play a critical role in detecting the early responses of post-resuscitation care and may predict in-hospital and long-term prognosis in patients affected by brain injury after cardiac arrest. In the future, this might optimize and individualize the treatment of post-cardiac arrest patients and potentially improve outcome.

## Surrogate end-point

The end-point defined as the LP ratio during ICU stay is a surrogate marker for poor neurological outcome and death. The surrogate marker is used as an alternative to a hard end-point in order to power this study adequately. Previously, LP ratio studies have been shown to be a reliable marker for poor outcome after traumatic brain injury [22–26]. Hence, our surrogate end-point reflects the clinically relevant question of whether higher MAP has a potential neuroprotective effect in our population.

## Strengths and limitations of this study

- Jugular bulb MD may provide a reliable global estimate of cerebral metabolic state and can be implemented as an entirely new and less invasive diagnostic tool for ICU patients after OHCA and has implications for early prognosis and treatment.
- This pragmatic trial is addressing the effect of higher MAP on global cerebral metabolism in patients resuscitated from OHCA.
- Randomized controlled trial design minimizes the risk of selection bias.
- Clinicians are not blinded to data obtained at the bedside from jugular bulb MD, so all outcome assessors will be blinded to minimize the risk of bias.
- Until now, no established gold standard method assessing global cerebral energy state has existed. Therefore, it is not possible to compare jugular bulb MD with an established method already in clinical use.

## Trial status

Protocol version 1.1. Date: March 8, 2018. The study is ongoing and currently enrolling. Recruitment began January 11, 2017, and is expected to be completed around January 8, 2020.

## Additional files

Additional file 1: SPIRIT (Standard Protocol Items: Recommendations for Interventional Trials) 2013 Checklist: Recommended items to address in a clinical trial protocol and related documents*. (PDF 144 kb)

Additional file 2: Informed consent. (PDF 183 kb)

## Abbreviations

CBF: Cerebral blood flow
CPC: Cerebral performance category
CT: Computed tomography
ICU: Intensive care unit
LP ratio: Lactate-to-pyruvate ratio
MAP: Mean arterial blood pressure
MD: Microdialysis
NIRS: Near infrared spectroscopy
OHCA: Out-of-hospital cardiac arrest
PAC: Pulmonary artery catheter
ROSC: Return of spontaneous circulation
rSO_2_: Regional oxygen saturation
SD: Standard deviation
SPIRIT: Standard Protocol Items: Recommendations for Interventional Trials
TTM: Targeted temperature management
