# Supplementary material for: Design paper of the “Blood pressure targets in post-resuscitation care and bedside monitoring of cerebral energy state: a randomized clinical trial”
Source: Trials. 2019 Jun 10;20:344. doi: 10.1186/s13063-019-3397-1 (PMC6558732; doi:10.1186/s13063-019-3397-1)
Supplement: Supplementary file 2 — Informed consent. (PDF 183 kb) [file 13063_2019_3397_MOESM2_ESM.pdf]

APPENDIX B

INFORMED CONSENT

Protocol Title: Blood pressure targets in post resuscitation care and bedside monitoring of cerebral energy state – a randomized clinical trial.

Study number: ClinicalTrials.gov Identifier: NCT03095742

Investigator name: Simon Molstrom, M.D., Ph.D.

Address: Odense University Hospital, Denmark

Consent Form:

The undersigned ____, born on the / /
in _____________________________________, mobile ____,
email address ____:

- legally acceptable surrogate agrees about participating in the study assessing the effect of different blood pressures levels on global brain metabolism in adult comatose out of hospital cardiac arrest (OHCA) patients.
- agrees that since blood pressure targets may be of importance in neuroprotection via mechanisms thought to reduce reperfusion injuries in the myocardium and in the brain, treatment allocation should be performed as soon as possible.
- agrees that randomization is performed immediately at admission before informed consent can be obtained according to the Declaration of Helsinki.

Information regarding processing of personal data:
According to the local law about processing of personal data, we are to provide the necessary information regarding the purposes and methods of processing of personal data and the scope of communication and diffusion, the nature of the data in our possession and how it is provided.

Aim
Jugular bulb microdialysis and immediate bedside biochemical analysis is in this study introduced as a new diagnostic tool to evaluate the effect of higher mean arterial blood pressure on global brain metabolism, and the degree of cellular damage after OHCA.

Transmission and distribution
Personal information will NOT be shared with third parties and will not be disseminated outside of the researchers of the study.

Consent
The undersigned (above) will be provided with written and oral information on this trial to be able to make an informed decision about participation in this trial.

Signature

____
